# Inflammation in the pathogenesis of depression: a disorder of neuroimmune origin

**DOI:** 10.1042/NS20220054

**Published:** 2023-07-13

**Authors:** Myles Corrigan, Aoife M. O'Rourke, Barry Moran, Jean M. Fletcher, Andrew Harkin

**Affiliations:** 1 Neuropsychopharmacology Research Group, School of Pharmacy and Pharmaceutical Sciences and Trinity College Institute of Neuroscience, Trinity College, Dublin, Ireland; 2 Transpharmation Ireland, Trinity College Institute of Neuroscience, Trinity College, Dublin, Ireland; 3 School of Biochemistry and Immunology, Trinity Biosciences Institute, Trinity College, Dublin, Ireland

**Keywords:** Depression, Inflammation, Neuroimmunology

## Abstract

There are several hypotheses concerning the underlying pathophysiological mechanisms of major depression, which centre largely around adaptive changes in neuronal transmission and plasticity, neurogenesis, and circuit and regional connectivity. The immune and endocrine systems are commonly implicated in driving these changes. An intricate interaction of stress hormones, innate immune cells and the actions of soluble mediators of immunity within the nervous system is described as being associated with the symptoms of depression. Bridging endocrine and immune processes to neurotransmission and signalling within key cortical and limbic brain circuits are critical to understanding depression as a disorder of neuroimmune origins. Emergent areas of research include a growing recognition of the adaptive immune system, advances in neuroimaging techniques and mechanistic insights gained from transgenic animals. Elucidation of glial–neuronal interactions is providing additional avenues into promising areas of research, the development of clinically relevant disease models and the discovery of novel therapies. This narrative review focuses on molecular and cellular mechanisms that are influenced by inflammation and stress. The aim of this review is to provide an overview of our current understanding of depression as a disorder of neuroimmune origin, focusing on neuroendocrine and neuroimmune dysregulation in depression pathophysiology. Advances in current understanding lie in pursuit of relevant biomarkers, as the potential of biomarker signatures to improve clinical outcomes is yet to be fully realised. Further investigations to expand biomarker panels including integration with neuroimaging, utilising individual symptoms to stratify patients into more homogenous subpopulations and targeting the immune system for new treatment approaches will help to address current unmet clinical need.

## Introduction

A role for the immune system has been implicated in the aetiology and pathophysiology of depression for many years [1]. Elevated concentrations of proinflammatory cytokines may be caused by pathogenic infection, auto-immunity, inflammation and psycho-physiological stress particularly when chronic, unpredictable and maladaptive. A compelling case for a role of immune-related mechanisms in depression is supported by the consistent finding of raised peripheral concentrations of proinflammatory cytokines in the blood of patients with depression [2]. Furthermore, patients receiving cytokine therapy for viral infections or as an oncotherapy are at an increased risk of suffering from severe depression-related symptoms including suicidal ideation [3]. A cytokine-induced depression may also arise from the mobilisation of innate and adaptive arms of the immune system. This is supported by the high frequency of comorbid depression with inflammatory and autoimmune disorders [4–6]. Elevated concentrations of proinflammatory cytokines is believed to impact (1) activation of innate and adaptive neuro-immune processes, (2) catecholaminergic and indoleaminergic neurotransmission, (3) kynurenine pathway mobilisation, (4) expression of neurotrophic receptors and neurotrophic signalling, (5) synaptic plasticity and neurogenesis and (6) regional brain connectivity. Subsequent sections will discuss how activation of the immune and inflammatory response system is hypothesised to impact these processes. A working hypothesis of the neuroimmune origins of depression is considered helpful in directing development of clinically relevant experimental models, in the development of targeted therapeutics and when informing clinical investigation.

## Cellular mediators of neuroimmune interaction

### Brain glia in depression pathophysiology

Microglia, astrocytes and oligodendrocytes each play an important role in depression pathophysiology. Recent directions as reported have focused on (1) characterising the role of neuroglial signalling in synaptic plasticity, (2) the interactive relationship between astrocytes and microglia in mediating neuroinflammation, and (3) the emergence of oligodendrocytes as key players in neuroinflammation and inflammatory related brain disorders.

### Microglia–neuronal interaction

Microglial signalling involving immune-related cellular mediators and receptors is believed to play a key role in synaptic plasticity [7]. For instance, Stellwagen & Malenka (2006) demonstrated in a series of electrophysiology experiments using wild-type and TNF-α-deficient glia and neurons that microglial-derived TNF-α modulates activity-dependent synaptic plasticity [8]. Furthermore, brain-derived neurotrophic factor (BDNF, a key neurotrophic factor that encourages growth and differentiation of neurons and formation of synapses) derived from microglia has been shown to regulate plasticity in neurons via the tropomyosin-related kinase receptor B (TrkB) [9]. Behaviourally, loss of microglial-derived BDNF in mice reduces rotarod performance improvement after motor training and fear response to a conditioned audio stimulus [9]. In addition, microglial interferon signalling is postulated to play a key role in cognitive function. Minter et al. (2016) showed that genetic deletion of the type 1 interferon receptor partially reversed cognitive deficits in the Morris water maze and attenuated microgliosis in a mouse model of Alzheimer’s disease [10]. These microglial signalling pathways thus offer potential targets for amelioration where a loss of plasticity and cognitive deficits feature.

Fractalkine signalling is a key mechanism whereby neurons communicate with microglia. This occurs via the neuronal chemokine CX3CL1, and its microglial receptor CX3CR1 [11], believed to be the neuronal ‘off’ signal that keeps microglia in their resting state [12]. CX3CR1-deficient mice have been used to investigate microglia–neuron cross-talk in response to immune stimulation and its effect on plasticity. Cardona et al. (2006) showed that CX3CR1-deficient mice were more vulnerable to neuronal loss following systemic LPS injection compared to wildtype [13]. CX3CR1-deficient mice were also resistant to the positive effects of environmental enrichment on hippocampal long-term potentiation [14]. Milior et al. (2016) showed that CX3CR1-deficient mice were unresponsive to chronic stress. CX3XR1-deficient mice subjected to unpredictable chronic mild stress were resistant to stress-induced reductions in saccharin preference indicative of a reduced anhedonic state, together with associated changes to microglia morphology and reductions in synaptic long-term potentiation [15]. Taken together, fractalkine signalling presents as a key mediator of microglial–neuronal communication and related modulation of neuronal plasticity with evidence suggesting stress and immune stimulation have differing consequences.

### Astroglia–microglial–neuronal cross-talk

As a common feature of depression is aberrant glutamatergic neurotransmission [16,17], astrocyte dysfunction is strongly implicated in its pathophysiology. The implication of astrocyte pathology in depression is supported by the work of Rajkowska, Stockmeier and colleagues. This group have found GFAP mRNA expression and GFAP immunoreactive astrocyte density to be decreased in the white matter of the ventral prefrontal cortex of depressed patients when compared with control subjects [18]. This supports an earlier study from the same group which found that astrocyte density in the hilus was significantly reduced in unmedicated depressed subjects when compared to healthy controls. This study also reported that GFAP-immunoreactive area fraction in the CA2/CA3 region of the hippocampus was negatively correlated with duration of depression in suicide victims [19]. These findings also support seminal work by Cotter et al. (2002) who found glial cell density to be reduced within brain regions associated with mood and behaviour in subjects with MDD when compared with healthy controls [20]. While evidence strongly implicates astrocyte pathology in depression, the astrocyte-specific cellular mechanisms that contribute to depression have remained elusive.

The ability of astrocytes to regulate microglial activity has increasingly come to light in recent years. Astrocyte-derived signalling molecules that have been shown to influence microglial activity include TGF-β [21], IL-33 [22] and orsomucoid-2 [23], amongst others. Norden et al. (2014) demonstrated a key role of astrocyte-derived TGF-β in attenuating LPS-induced microglial activation. Their study showed that IL-10 augmented the expression of TGF-β in LPS-stimulated astrocytes and that astrocyte-derived TGF-β modulated the LPS-induced microglial activation state by reducing IL-1β and IL-6 expression and enhancing expression of CX3CR1 and the IL-4 receptor α [21]. Inhibition of TGF-β signalling in mice caused exaggerated sickness behaviour and increased coronal brain mRNA levels of IL-1β, IL-6, TNF-α and CD14 post-LPS challenge [21], highlighting the importance of IL-10-induced astrocyte-derived TGF-β in microglial activation. While the *in vivo* element of this study is perhaps limited by a small sample size, the findings indicate a bi-directional relationship between microglia and astrocytes. The finding is also supported by results of a more recent study which found that disrupted TGF-β/IL-10 in astrocytic IL-10ra^KO^ mice led to increased social avoidance and higher microglial IL-1β and TNF mRNA expression in response to peripheral lipopolysaccharide [24]. These results are further supported by the work of Zhang et al. (2020) who found that blocking astrocytic TGF-β *in vitro* led to a reduction of microglial ramification (a marker of the microglial resting state) [25].

The sequence of microglial and astrocyte activation has also been the subject of multiple studies. One study compared the time course of sickness behaviour and glial cytokine expression post-LPS challenge in mice [26]. Sickness behaviour measured by reduced social interaction and locomotion was almost immediate and resolved within 48 h. Peak microglial expression of IL-6, IL-1β, TNF-α, CCL2 and IL-10 occurred at 2–4 h post-LPS, while peak astrocyte expression of IL-1β, CCL2 and TNF-α occurred 12 h post-LPS injection [26], suggesting that following an immune challenge, microglia activation precedes and perhaps induces astrocyte activation. This hypothesis is further supported by the work of Liddelow et al. (2017) who found that IL-1α, TNF-α and complementary component 1q, which are secreted by activated microglia is sufficient to induce a neurotoxic reactive phenotype in neighbouring astrocytes [27]. The data presented in these studies underline the importance of astrocyte–microglia interaction activation of one cell type can influence the other.

### TNF-α signalling in glial–neuronal interaction

Astrocytic regulation of neuronal plasticity has been demonstrated in recent years. Habbas et al. (2015) demonstrated that TNF-α results in increased glutamate release at excitatory hippocampal synapses leading to altered excitability of hippocampal granule cells [28]. TNF-α causes activation of astrocytic TNF receptor 1 (TNFR1) which in turn triggers an astrocyte–neuron signalling cascade which modifies hippocampal excitatory synapses [28]. Astrocytic TNF-α signalling is also reported to affect cognitive function which may be of relevance to multiple sclerosis [28], Alzheimer’s disease [29], and depression [30]. A comprehensive meta-analysis conducted by Patlola et al. (2023) reported that cognitive function was inversely proportional to systemic concentrations of TNF-α (along with IL-6, IL-1β and CRP) in patients with schizophrenia [31]. A meta-analysis by Köhler et al. (2017) reports that peripheral concentrations of TNF-α were elevated in patients with depression [32]. Conversely, Ng et al. (2018) reported no difference in peripheral concentrations of TNF-α in aged, depressed patients or patients with Alzheimer’s disease when compared with healthy controls in their meta-analysis [33], suggesting that alterations in peripheral TNF-α seen in depression may be age-dependent.

TNF-α has also been associated with cognitive dysfunction and various depressive symptoms in multiple animal studies [34]. For example, Şahin et al. found that chronic treatment of rats subjected to chronic unpredictable mild stress with TNF-α inhibitor infliximab prevented stress-induced cognitive deficits [35]. Additionally, Klaus et al. (2016) studied the behavioural response of TNF-α administration in various brain regions using an adenovirus-associated viral TNF-α vector (AAV-TNF) and compared each response with TNF-α administration to the periphery (IP-TNF) [36]. Delivery of AAV-TNF into the lateral ventricle increased anxiety behaviour, while delivery into the amygdala reduced motivation and increased anxiety [36]. Finally, AAV-TNF delivery into the hippocampus led to a reduction in body weight and an increase in conditioning to (but not memory of) an aversive stimulus [36]. While it is clear that TNF-α signalling in specific brain regions mediates diverse behavioural responses, future studies are likely to refine existing knowledge of the role of TNF signalling with regard to specific depression symptoms.

### An emerging role for oligodendrocytes

An area of growing interest is the elucidation of the role of oligodendrocytes in neuroinflammatory related brain disorders including depression. Myelination and oligodendrocyte pathologies are often associated with neuropsychiatric conditions where psychosis and cognitive dysfunction feature [37]. Cathomas et al. (2019) demonstrated that chronic social defeat in mice (a popular animal model of stress and depression) resulted in the down-regulation of various oligodendrocyte-related genes encoding myelin and myelin–axon-integrity proteins [38]. Mice deficient in the oligodendrocyte gene *Cnp1* exhibited reduced social interaction when compared to wild-type mice [38]. Furthermore, Poggi et al. (2022) showed that *Cnp1* deficiency increased stress-induced microglia activation [39]. Social defeat increased densities of cells positive for oligodendrocyte markers (CC1 and aspartoacylase) in the amygdala [39]. Social defeat also reduced proliferative oligodendrocyte precursor cells in the basolateral amygdala and medial prefrontal cortex – key limbic structures implicated in depression [39]. In the medial prefrontal cortex, defeat increased myelin basic protein integrated density and myelin thickness [39]. Further study is required to establish if these changes could contribute to aversive learning and memory that occur following chronic social defeat and moreover in human neuropsychiatric disorders where stress is a contributing aetiological factor.

### Brain endothelia–microglial interactions and recruitment of peripheral leukocytes

Due to the blood–brain barrier, the CNS has historically been regarded as immune-privileged. Recently, however, evidence indicates that brain endothelia respond to microglial/inflammatory-related signals by recruiting peripheral leukocytes and trafficking them into the CNS (see Table 1) [40]. Trafficked leukocytes have been implicated in depression pathophysiology, particularly in post-mortem brain of patients who die by suicide [41]. Mechanistically, activation of microglia by stress or immune challenge induces proinflammatory cytokine and chemokine expression. These factors can interact with endothelial cells in the blood–brain barrier, causing them to recruit immune cells from the periphery which exacerbate neuroinflammation [42]. IL-1β signalling and cell adhesion molecules are heavily implicated in this microglia–endothelia-periphery cross-talk [43–46]. McKim et al. (2018) showed that depletion of microglia prevents stress-induced recruitment of monocytes into the brain [43]. Disruption of IL-1β signalling in monocytes by genetically knocking out caspase-1 (the enzyme that cleaves IL-1β into its active form) in these cells prevented stress-induced monocyte migration into the brain [43].

**Table 1:** A role for brain endothelia and trafficked immune cells from the periphery in the mouse repeated social defeat model of stress and depression

Main findings	Reference
**●** Activation of brain regions involved in threat appraisal which coincided with an increase in microglial activation, an increase in adhesion molecule expression in brain endothelial cells and deficits in the open field test **●** Recruited monocytes adhered to IL-1R1-expressing brain endothelial cells which was dependent on microglia **●** Monocyte cell adhesion molecules (CAM; namely VCAM-1 and ICAM-1) were up-regulated in brain endothelium cells following social defeat **●** Treatment with the microglia inhibitor (minocycline) blocked these effects, as well as reducing deficits in the open field test **●** Blocking stimulating factor 1 receptor (CSFR1) with plexxikon 5622 decreased stress-induced microglial activation, brain macrophages and deficits in the open field test	[43]
**●** Vascular cell adhesion molecule 1 (VCAM-1) and intercellular adhesion molecule 1 (ICAM-1) were up-regulated in an exposure-dependent manner to repeated social defeat. This was confined to the prefrontal cortex and the paraventricular nucleus	[44]
**●** IL-1R1 knockout mice did not have increased circulating myeloid cells following repeated social defeat, leading to limited macrophage trafficking **●** Global IL-1R1 expression is essential for macrophage migration into the brain **●** Local deletion of IL-1R1 in brain endothelial cells rescued defeat-induced increases in expression of IL-1β, TNFα and IL-6 and deficits in the open field, and light–dark preference tests **●** Local deletion could not prevent stress-induced recruitment of peripheral macrophages into the brain and activation of microglia	[45]
**●** Defeat induced monocyte trafficking, and microglial activation and increased hippocampal expression of IL-1β, TNFα, IL-6 and vascular endothelial growth factor (VEGF) **●** Minocycline attenuated stress-induced microglia activation and associated monocyte trafficking in the dentate gyrus of the hippocampus	[47]
**●** Defeat induced increased macrophage recruitment into the brain, increased social avoidance and open field test deficits **●** Changes in macrophage trafficking and social avoidance did not persist at 24 d but these changes were re-established by a sub-threshold social defeat in stress-sensitised mice	[48]
**●** Defeat induced deficits in the open field test were associated with egress of Ly6C^hi^ monocytes from the spleen. **●** Splenectomy prior to repeated social defeat prevented the re-establishment of monocyte trafficking and open field deficits in mice that were sensitised to stress **●** Peripheral sympathetic inhibition using guanethidine blocked re-establishment of monocyte trafficking and open field deficits in mice that were sensitised to stress	[49]

Sympathetic nervous system and hypothalamic–pituitary–adrenal (HPA) axis activation in response to stress promotes the release of monocytes from the bone marrow into the circulation which are subsequently redirected towards the CNS depending on microglial activation [42]. McKim et al. (2016) identified the spleen as a key reservoir of primed monocytes in the mouse repeated social defeat model [49]. Peripheral sympathetic inhibition using guanethidine (a catecholamine release inhibitor) disrupted monocyte trafficking which also rescued stress-induced deficits in the open field test [49]. The work of Sheridan and colleagues has extensively investigated the relationship between repeated stress, neuronal activation and the immune response. This group propose that stress sensitisation is a result of neuronal activation in fear and threat appraisal centres of the brain leading to microglial activation in corresponding regions. Activated microglia are believed to then communicate with brain endothelia to recruit peripheral monocytes into the brain and exacerbate existing neuroinflammation, contributing to an exaggerated behavioural and immune response to subsequent subthreshold stressors [42,50–53].

Animal models of stress and social defeat paradigms in particular have been invaluable in assessing such mechanisms. Typically applied to rodents, this protocol subjects animals to multiple bouts of social defeat to larger, more aggressive counterparts. Animals that have suffered multiple bouts of social defeat typically exhibit a depressive phenotype, which includes social avoidance, decreased motivation and metabolic and weight disturbances [54]. The repeated social defeat model has also been shown to increase expression of IL-1β, CCR2, CXCR2, TNF-α and TLR4 in the CNS [55] and is valuable in the study of glial–neuronal interactions. While many variations in the protocol exist, social defeat paradigms as a whole are reliable and offer a robust model to study various stress-related psychopathologies such as in anxiety, post-traumatic stress disorder and depression.

## A role for adaptive immune cells in the neuroimmune origins of depression

**Figure 1:**
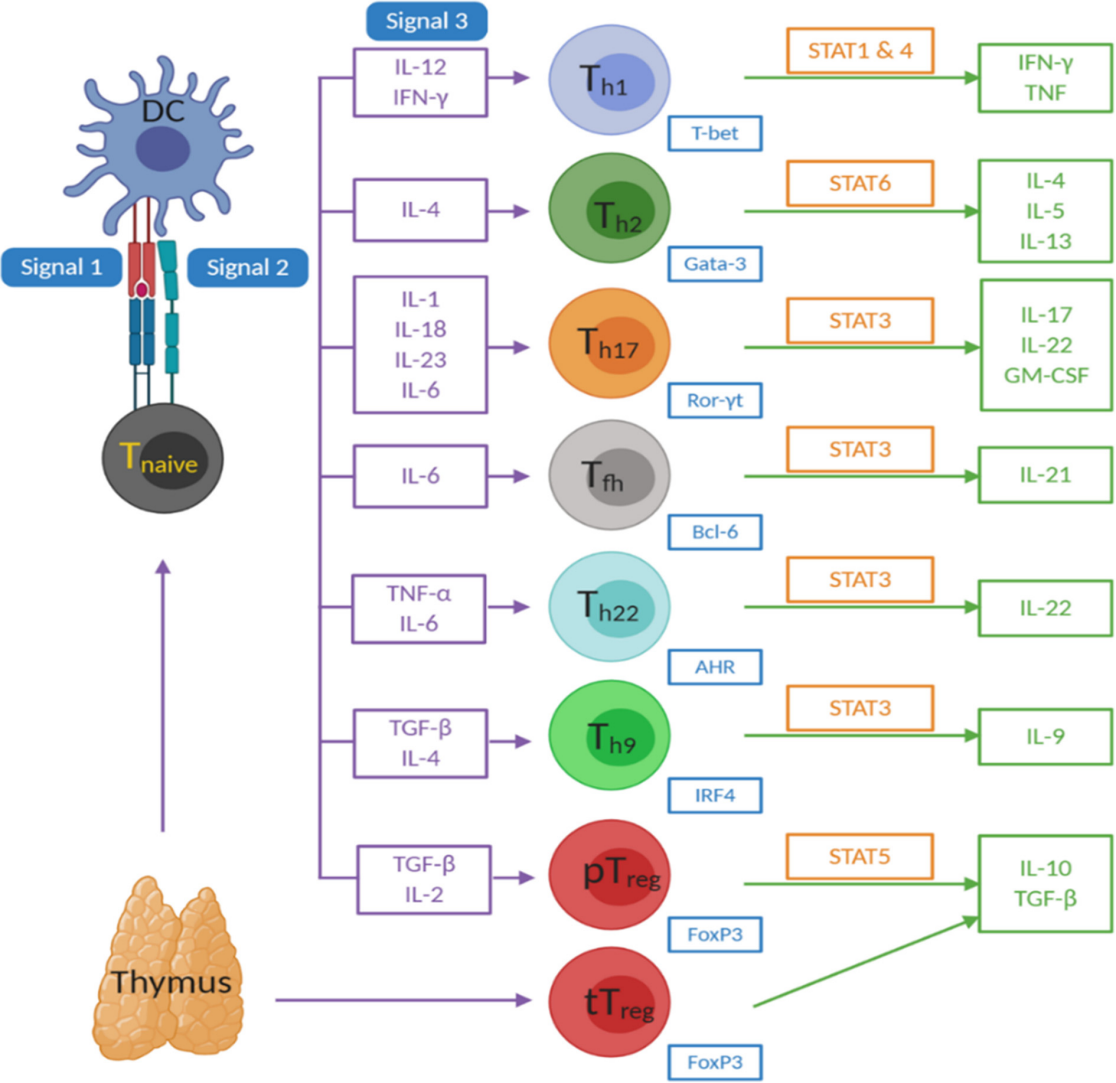
Differentiation of CD4 effector T-cell subsets Upon engaging an infectious agent, dendritic cells (DC) may become activated via their pathogen recognition receptor and phagocytose the pathogen, presenting antigens via the MHC class II pathway. Naïve T-cell activation is initiated through engagement of this antigen: MHC II complex with the T-cell receptor (signal 1), along with co-stimulation through CD28 (T cell) and CD80/86 (antigen presenting cell; APC) binding (signal 2). Upon activation, Th cells undergo clonal expansion and differentiate into one of seven effector subsets (Th1, Th2, Th17, Tfh, Th22, Th9 and pTreg cells) depending on the cytokines present in their microenvironment (signal 3). Signalling induced by these polarising cytokines activates specific STATs and transcription factors, which direct the function of the Th cell type, including production of their signature cytokines. Peripheral Treg cells have a similar phenotype and function to thymic-derived Treg cells, but thymic derived cells do not require APC engagement. Created with BioRender.com.

A role for innate immunity implicating activated microglia and circulating monocytes in the pathophysiology of depression has been previously reviewed [56–61]. Perhaps the most significant strides in depression research over the past decade have been in elucidating a role for the adaptive immune system in depression aetiology and pathophysiology (for review, see Beurel, Medina-Rodriguez & Jope (2022) [62]). Conventional T cells and B cells make up the cells of the adaptive immune system. B cells (or B lymphocytes) mediate the production of antigen-specific immunoglobulin (Ig), whilst T lymphocytes carry out the cell-mediated responses. Helper T (Th) cells, characterised by CD4 expression, play a pivotal role in mounting a robust, antigen-specific immune response to invading pathogens. Th cells become activated upon engagement of their T-cell receptor with antigens mounted on major histocompatibility complex (MHC) class II molecules [62] on antigen-presenting cells, along with co-stimulation with CD28 and CD80/86. Once antigen-experienced, T cells differentiate into subsets depending on the cytokines they are exposed to (see Figure 1). Th1, Th2, Th17 cells and regulatory T (Treg) cells are the most studied Th cell subsets in depression research. Th1 cells differentiate in the presence of interferon-γ (IFN-γ) and interleukin (IL)-12, Th2 cells with IL-4, Th17 cells with IL-1, IL-18, IL-23 and IL-6, and peripheral Treg cells with TGF-β and IL-2. Thymic-derived Treg cells do not require antigen-recognition or cytokine stimulation. Th1 cells express IFN-γ as their signature cytokine, Th2 cells IL-4, IL-5 and IL-13, Th17 cells IL-17, IL-22 and GM-CSF, and Treg cells IL-10 and TGF-β. Th1 and Th17 cells have play a pathogenic role in many autoimmune diseases, whilst Treg cells are considered to play a protective role in autoimmunity, dampening aberrant inflammatory responses. Multiple studies have indicated abnormal numbers of Th cells in depressed patients, with altered frequencies of Th17 and Treg cells implicated in depression pathophysiology. Underlying mechanisms that may lead to dysregulated T-cell populations in depression could involve stress-induced glucocorticoid signalling, sympathetic nervous system activation and irregularities in the gut microbiome [62]. Patas et al. (2018) found T cells in patients with depression to have significantly lower CXCR3 and CCR6, important receptors in the regulation of T-cell trafficking and differentiation [63].

### Th17, Th1 and Th2 cells

Chen et al. (2011) found a higher serum level of IL-17, higher number of inflammatory Th17 cells, and a lower number of Treg cells in patients with MDD, resulting in a higher Th17/Treg cell ratio [64]. Similarly, Ghosh et al. (2020) found that unmedicated patients with MDD have a higher percentage of Th17 cells and a higher Th17/Treg cell ratio. Furthermore, IFN-γ^+^ Th17 cell cultures from depressed patients secreted more IL-17 compared with healthy controls [65]. Schiweck et al. (2020) reported that patients judged to be at high risk for suicide had the highest levels of peripheral Th17 cells and memory T cells [66]. Nadeem et al. (2017) investigated the role of IL-17 in depressive behaviours in mice using an imiquimod model of psoriatic inflammation. IL-17 and Th17 cells are strongly implicated in psoriasis, and the authors showed that inflammation led to enhanced IL-17A (the archetypal IL-17 member) expression in peripheral immune cells, with associated increases in NF-κB/p38 MAPK signalling and depressive-like behaviours [67]. Furthermore, treatment with an anti-IL-17A monoclonal antibody prevented the development of imiquimod-induced depressive behaviours [67]. Th17 cells were similarly implicated in generalised anxiety disorder where isolated T cells from patients exhibited lower proliferation following activation *in vitro* compared with healthy controls [68]. The individuals with anxiety also had lower Th1 and Th2 cells, with more adopting a Th17 phenotype. This study also demonstrated that dopamine was more effective in modulating T-cell cytokine production in healthy controls than in individuals with anxiety [68]. Beurel, Harrington & Jope (2013) demonstrated that mice subjected to learned helplessness or chronic restraint stress had higher levels of Th17 cells in their brain [69]. Administration of Th17 cells to Rag2^−/−^ mice (mice lacking T cells) led to increased susceptibility to learned helplessness [70]. Th17 cells administered to wild-type mice accumulated in the hippocampus of learned-helpless mice, exhibiting pathogenic and follicular characteristics with increased endogenous T cell differentiation [70]. Ambrée et al. (2019) found that mice that are susceptible to social defeat had higher numbers of Th17 cells in the spleen, with less Treg cells and lower serum TGF-β concentrations [71].

### Treg cells

There are varying reports concerning Treg cell levels in depression (Table 2). Jahangard & Behzad (2020) found that the number of Treg cells (characterised as CD4^+^CD25^hi^ cells, which may include a small number of activated non-Treg cells) in untreated depressed patients were diminished when compared with healthy controls [72]. This was similar to findings by Grosse et al. (2016) who reported decreased Treg cells (here characterised by CD4^+^CD25^hi^ and the Treg cell transcription factor FoxP3^+^) in MDD patients when compared with healthy controls [73]. By contrast, Suzuki et al. (2017) reported that MDD patients had an increased number of (CD127^lo^/CCR4^+^) Treg cells [74]. Kim et al. (2012) found that depletion of Treg cells in mice using an anti-CD25 antibody led to depression and anxiety related behaviours in the forced swim and elevated plus maze tests, respectively [75]. This was associated with an up-regulation of various proinflammatory cytokines. This study also presented evidence that Treg cell depletion is associated with a reduction in serotonin concentrations within the hippocampus of non-stressed anti-CD25 treated mice compared with non-stressed control mice. The disparity in reports of Treg cell measures in MDD may in part arise from the different gating strategies used in flow cytometry to define the cells; ranging here from CD4^+^CD25^hi^, to CD161^lo^CCR4^+^, to CD3^+^CD4^+^CD127^lo^CD25^hi^FoxP3^+^. Similarly, anti-CD25 antibodies will deplete some activated T cells in addition to Treg cells.

**Table 2:** Evidence implicating the adaptive immune system in depression

Focus of study	Findings	Reference
***Evidence for the role of T cells***		
Antidepressant-naïve MDD patients	- MDD patients had: ​ ● Lower T-cell surface expression of the chemokine receptors CXCR3 and CCR6 ​ ● Higher frequency of CD4^+^CD25^high^CD127^low/−^ cells ​ ● Increased *FOXP3* mRNA expression in purified CD4+ T cells ​ ● Less diverse library of CD4+ T cells	[63]
Antidepressant-naïve MDD patients	- MDD patients had: ​ ● Higher frequency of serum antinuclear antibodies ​ ● Increased number of peripheral Th17 cells ​ ● Decreased number of Treg cells ​ ● Higher mRNA expression of RORγT in peripheral blood lymphocytes ​ ● Higher serum IL-17 concentration	[64]
Antidepressant-naïve (first episode) MDD patients	- MDD patients had: ​ ● Higher percentage of Th17 cells ​ ● No significant difference in percentage of Treg cells ​ ● Higher Th17/Treg cell ratio ​ ● Increased secretion of IL-17 in IFNγ + Th17 T-cell subsets	[65]
Antidepressant-naïve MDD patients	- MDD patients had: ​ ● Increased Th1/Th2 cell ratio in peripheral blood ​ ● Decreased number of Treg cells ​ ● Reduced expression of the 5-HT_1A_ receptor in Treg cells	[76]
Untreated and treated MDD patients	- Untreated MDD patients had: ​ ● Lower frequencies of FOXP3 and pSTAT5 in peripheral Treg cells when compared with healthy control and SSRI-treated patients ​ ● Higher *in vitro* proliferation of CD4^+^ T cells when compared with healthy control - MDD patients in general had: ​ ● Similar numbers of CD45RA-expressing Treg cells as healthy controls	[72]
MDD patients at least three weeks treatment-free	- MDD patients had: ​ ● Increased percentage of CD127^low^/CCR4^+^ Treg cells ​ ● Increased percentage of memory Treg cells ​ ● Reduced number of CD56^+^CD16^−^ NK cell counts	[74]
Antidepressant-naïve MDD patients prior to treatment with either venlafaxine or imipramine	- MDD patients had reduced percentage of Treg cells - Antidepressant treatment resulted in increases in Treg cell number - Antidepressant non-responders had a higher baseline percentage of CD8^+^ cytotoxic T cells and decreased percentage of NK cells	[73]
MDD patients (antidepressant treatment permitted)	- MDD patients had: ​ ● Reduced percentage of NK cells ​ ● Increased percentage of B and T cells ​ ● Increased percentage of memory T helper cells - MDD patients with a high suicide risk had: ​ ● Increased percentage of Th17 cells compared with all other risk groups ​ ● Increased percentage of memory T helper cells compared with low and medium risk groups	[66]
Combining medications to enhance depression Outcomes (CO-MED) clinical trial participants	- Higher baseline IL-17 (Th17 marker) was predictive of a greater reduction of depression severity in the bupropion-SSRI treatment group - There was an IL-17 × treatment interaction effect for depression severity	[77]
*In vitro* assays of cells derived from MS patients with co-morbid depression (MS/MDD)	- MS/MDD patients had: ​ ● Higher proliferation and Th17-related cytokine production in CD4^+^ and CD8^+^ T-cell cultures in response to TLR2 and TLR4 (but not TLR5 or TLR9) agonists ​ ● Reduced IL-10 release in response to TL4 stimulation in *in vitro* cultures ​ ● Higher TLR2 and TLR4 expression on CD4^+^ and CD8^+^ T-cell surfaces - Treatment of patients with SSRI or in vitro addition of an SSRI resulted in reduced production of Th17-related cytokines in response to agonism of TLR2 and TLR4	[78]
Mice subjected to (1) stress paradigms, (2) administration of exogenous T cells or (3) depletion of the RAR-related orphan receptor γ transcription factor (RORγT; drives Th17 cell differentiation)	- Brain levels of Th17 cells were elevated by chronic restrain stress and learned helplessness - Mice who were administered Th17 cells developed learned helplessness. In comparison, vehicle-treated mice did not - Th17 cell-treated mice exhibited impaired feeding and social interaction behaviours - Mice with deficiency of RORγT were resistant to learned helplessness - Inhibition of RORγT or treatment with anti-IL17A antibodies supressed T-cell function and reduced Th17-dependent learned helplessness	[69]
Mice subjected to (1) administration various T-cell types and/or (2) depletion of endogenous T and B cells	- Th17 (but not Th1 or Treg) cell administration increased susceptibility to learned helplessness in mice devoid of B and T cells. These cells accumulated in the hippocampus - Hippocampal Th17 cells from learned helplessness mice expressed CCR6, IL-23R, CXCR5 and PD-1	[70]
Mice subjected to social defeat stress	- Mice susceptible to social defeat stress had: ​ ● Lower T-cell frequencies ​ ● Increased IL-17-producing CD4^+^ and CD8^+^ T cell numbers in the spleen ​ ● Reduced numbers of Treg cells ​ ● Reduced expression of TGF-β	[71]
Mice subjected to anti-CD25 antibody administration	- Mice treated with anti-CD25 antibody showed depression and anxiety related behaviours in the forced swimming and elevated plus maze tests, respectively	[75]
Mice with a T- and B-cell deficiency	- Mice deficient in T and B cells had impaired hippocampal neurogenesis	[79]
IL-17 knockout mice	- Mice deficient in IL-17 (Th17 cell marker) showed enhanced neurogenesis in the dentate gyrus of the hippocampus	[80]
Healthy males undergoing brief mental stressor	- Stressor resulted in a decrease in the number of Treg cells as well as naïve and central memory T cells - Β_1_-adrenergic and glucocorticoid α receptors were overexpressed in Treg cells - This is a little out of place here - I suggest you remove this from the table and refer to in the text under HPA and SAM axis below	[81]
MDD patients	- MDD patients had a higher number of HLADR^+^ and CD19^+^ B cells - MDD patients had a higher percentage of HLADR^+^ and CD21^+^ B cells - Melancholia patients had a higher percentage and number of CD21^+^ and CD19^+^ B cells	[82]
Medicated MDD patients	- Severely depressed patients had reduced frequencies of naïve lgD^+^CD27^−^ memory B cells - MDD patients had reduced CD1d^+^CD5^+^ and CD24^+^CD38^hi^ transitional B cells - Depression severity was associated with CD5 surface expression on transitional B cells, which was normalised b antidepressant treatment	[83]
Medicated and unmedicated MDD patients	- There was no difference in CD19^+^ B cells between patients and healthy controls	[84]
Patients with systemic lupus erythematosus (SLE)	- Co-morbid psychosis and/or depression in SLE patients was associated with autoantibodies against the ribosomal P protein (anti-P)	[85]
Patients with systemic lupus erythematosus (SLE)	- 64.7% of SLE patients with psychosis and mood disorders had antibodies against endothelial cells (AECA) compared to 29.4% of SLE patients without psychosis and mood disorders - No correlation was found between psychiatric disorders and autoantibodies against cardiolipin, β2 glycoprotein I, Ro, Ro52, La, glial fibrillary acidic protein, ribosomal P protein, dsDNA or nucleosomes in this patient group	[86]
Patients with systemic lupus erythematosus (SLE)	- Serum antibodies against the NMDA receptor were associated with depressive mood in a SLE patient sample	[87]
Mice injected with anti-P	- Mice injected with anti-P had increased depressive behaviour as indicated by increased immobility in the forced swimming test	[88]

### T-cell neuronal interaction

T cells are believed to interact in central processes including neurotransmission and in particular excitatory and serotonergic, and neurogenesis [62]. Sales et al. (2021) investigated the effect of serotonin and antidepressants on Th17 cell differentiation [78]. This study found that production of Th17 cell-related cytokines upon stimulation was higher in T cell cultures derived from depressed multiple sclerosis patients when compared to non-depressed multiple sclerosis patients [78]. Furthermore, cytokine production was attenuated when T cells were treated *in vitro* with serotonin, or when depressed patients were treated with selective serotonin inhibitors (SSRIs) [78]. Jha et al. (2017) found that higher concentrations of IL-17 prior to combination bupropion-SSRI treatment was associated with a greater reduction in depression severity following treatment, indicating that IL-17 and Th17 cell numbers may serve as an effective biomarker for antidepressant response [77]. Kostic et al. (2017) assessed the effect of IL-17A on glutamate processing by astrocytes *in vitro* [89]. At certain concentrations, IL-17A reduced the expression of glutamate transporters and glutamine synthetase in rodent-derived astrocytes. They also reported that IL-17A stimulated Ca^2+^-dependent glutamate release in a dose-dependent manner. Taken together, these findings suggest that IL-17A can influence glutamate transmission by reducing astrocyte ability to take up and convert glutamate to glutamine, as well as enhancing astrocytic glutamate release [89].

In terms of the relationship between adaptive immunity and neurogenesis, Ziv et al. (2006) found that T cells are necessary for hippocampal neurogenesis induced by environmental enrichment [79]. Using transgenic mice deficient in T and B cells, they found that neurogenesis in the hippocampus was impaired and could not be enhanced by environmental enrichment. This was accompanied with lower BDNF immunoreactivity in the immunodeficient mice [79]. Whilst T cells as a whole are necessary for neurogenesis, the subpopulation of Th17 cells may in fact reduce neurogenesis. IL-17 knockout mice had increased levels of neurogenesis in the dentate gyrus of the hippocampus [80]. The IL-17 knockout mice also had lower hippocampal levels of proinflammatory cytokines such as IFN-γ, TNFα, IL-1β and IL-6 [80].

### T cells and antidepressant response

Analysis of T-cell populations in MDD patients may play a role in providing information that can help predict and assess a patient’s response to a specific antidepressant therapy (e.g. SSRI). One study reported that although Treg cell levels did not predict clinical outcome, antidepressant treatment lead to a pronounced increase in Treg cell populations [73]. It may be that antidepressants reinstate normal cytokine levels by increasing Treg cell population. It should be noted that Treg cells express both serotonergic receptors [76] and adrenergic receptors [81] which could provide a potential mechanism, whereby antidepressants modulate Treg cell levels and activation. Although not discussed further here, higher levels of CD8^+^ cytotoxic T cells and decreased natural killer cells were observed at baseline when compared with antidepressant responders [73].

### B cells

B cells also play a crucial role in the adaptive immune response and are required for both antibody production and T-cell activation. B cells can be activated by foreign peptides directly, or indirectly by antigen presenting cells such as dendritic cells (Figure 1). B cells act as antigen presenting cells themselves in secondary lymphoid organs, whereby they sample antigen through their B-cell receptor, then process and present the antigen via MHC-II to naïve T cells, thus mounting an immune response specific to that antigen. Th cell-derived cytokines may then act on the B cell to enhance its survival and proliferation, as well as regulating the type of antibody produced.

As with T cells, there is conflicting information on the role of B cells in MDD (see Table 2), with reports of increased B cell infiltration to the brain parenchyma of patients with mood disorders [90], increased circulating MHC-II^+^ B cells in subsets of MDD patients [82], reduction in circulating Breg cells and B naïve cells but not in B memory cells [83] as well as reports of no differences compared with healthy controls [84]. Damage-associated molecular patterns (DAMPs), particles derived from tissue damage and cell death processes, may also play a pathogenic role in MDD. Stress-induced DAMPs may both initiate or contribute to existing inflammation. Uric acid [91,92], ATP [93] and heat shock protein 70 (HSP70) [94], all of which activate an inflammatory response, have been implicated in MDD patients or in preclinical models of stress and depression [95–99]. Furthermore, induction of HSP72 expression following heat stress and spinal cord injury in mice is prevented by serotonin synthesis inhibitor p-CPA [100]. Self-antigen reactive (autoreactive) T and B cells can lead to the development of autoimmune conditions. Interestingly, MDD and other psychiatric disorders are more prevalent in patients with autoimmune disorders including systemic lupus erythematosus [101], rheumatoid arthritis [102] and multiple sclerosis [103]. Levels of autoantibodies in peripheral blood are associated with MDD; higher levels of autoantibodies including anti-P ribosomal autoantibodies [85], anti-endothelial cell autoantibodies [86] and NMDA receptor autoantibodies [87] have been found in patients with these autoimmune conditions who also experienced neuropsychiatric symptoms, compared with patients without symptoms. Furthermore, intracerebroventricular injection of affinity purified human anti-ribosomal P antibodies in mice induced depressive behaviours, while blocking the antibody improved their behaviours [88].

Each B-cell subset is capable of regulatory functions (Breg cells) – the expression of CD1 and CD5 is associated with their phenotype [104–106]. Increased expansion of Breg cells can reduce B-cell reactivity thus promoting tolerance. According to Ahmetspahic et al. (2018) MDD patients have decreased CD5 expression [83]. An absence of B-cell regulation may exacerbate the effects of autoreactivity associated with the condition. Overall, the alteration in B-cell populations indicates an environment in which uncontrolled reactivity takes place, aligning with the increase in Th17 cells and higher levels of inflammatory cytokines in MDD patients. However, given that many reports are conflicting, larger-scale coherent studies are required to clarify the role of T and B cells in depression.

## Neuroimmune mechanisms associated with predisposing factors to depression

Genetic predisposition, early development and environmental factors all play important causative roles in depression. The following subsections refer to these factors with respect to underlying pathophysiological mechanisms of neuroimmune origin in depression susceptibility.

### Genetic pre-disposition

While a single causative genetic mutation for immune-related depression does not exist, it is likely that a large number of genes, each with small additive contribution, can govern genetic predisposition to the disorder [107]. Genome-wide and twin-/family-based association studies estimate the heritability of depression to be in the region of 31–42% [108]. Additionally, there is consistent evidence that concordance of major depression is higher in monozygotic than in dizygotic twins [109,110]. Variations in immune-related genes are associated with depression and are believed to contribute to manifestation of the disorder. The most studied of such genes include those encoding IL-1β, IL-6, IL-10, monocyte chemoattractant protein-1, TNF-α, C-reactive protein and phospholipase A2 [111]. While a detailed discussion on these genetic variations is beyond the scope of this text, the reader is directed to Barnes, Mondelli & Pariante (2017) for a comprehensive review [111]. Evidence also indicates a gene × environment interaction that contributes to the pathogenesis of psychiatric disorders. For example, Caspi et al. (2003) showed that stressful life events were more likely to cause depression and suicidal tendencies in individuals with one or more copies of the short allele of the 5-HT T promoter region than individuals homozygous for the long allele [112].

### A role for the HPA and SAM axes

**Figure 2:**
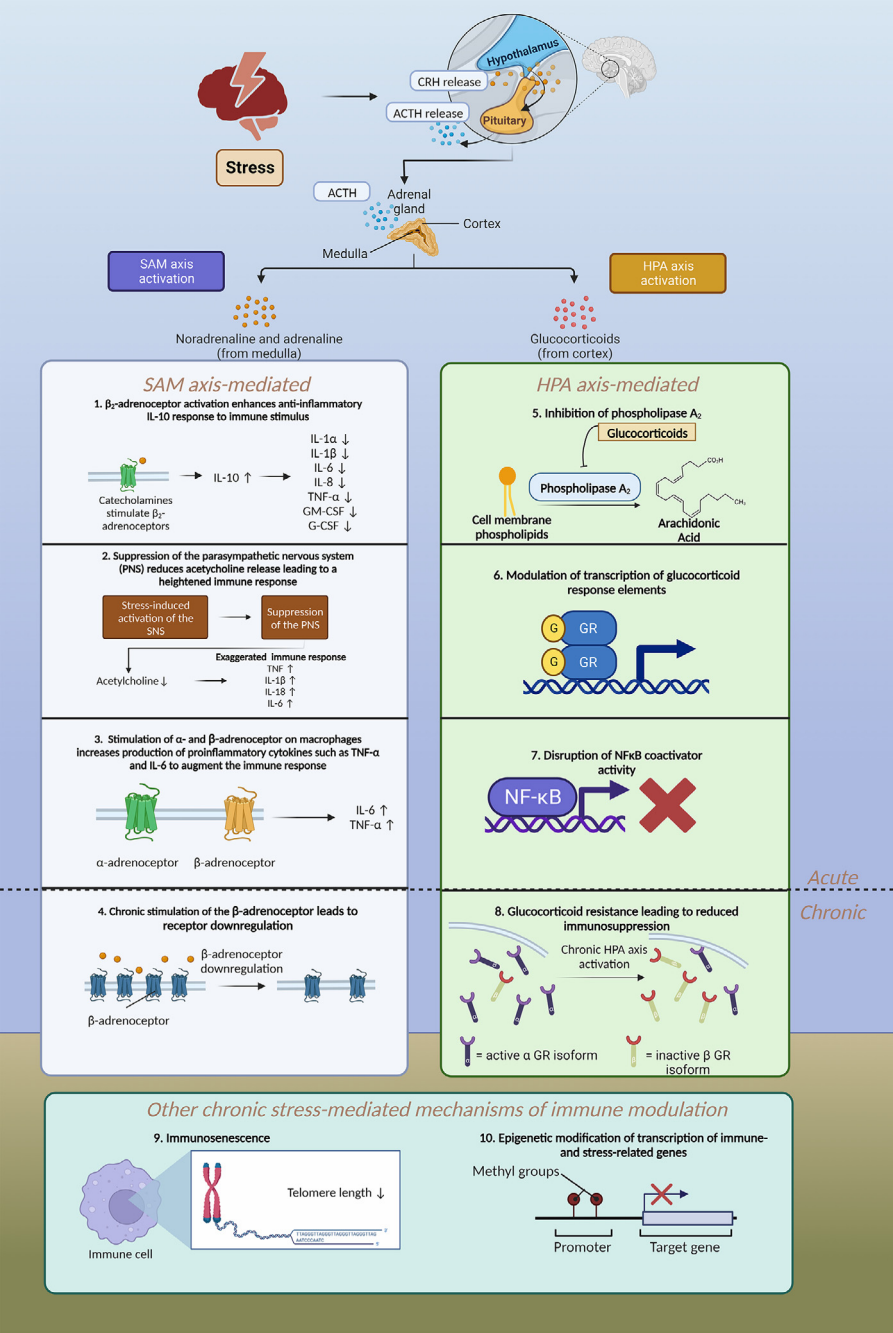
The relationship between stress and immune function Stress stimulates the HPA axis causing the hypothalamus to secrete corticotrophin-releasing hormone (CRH). This in turn stimulates the release of adrenocorticotropic hormone (ACTH) from the pituitary gland into the bloodstream. ACTH stimulates the release of glucocorticoid hormones from the adrenal cortex and adrenaline from the adrenal medulla. Stress stimulates the release of noradrenaline from sympathetic nerve endings. Noradrenaline and adrenaline released via SAM axis activation influence immune function. (1) Stimulation of the β-adrenoceptor leads to increased release of anti-inflammatory IL-10 [122]. IL-10 has an anti-inflammatory effect by suppressing synthesis of IL-1α, IL-1β, IL-6, IL-8, TNF-α, GM-CSF and G-CSF [123]. (2) Acetylcholine has been shown to have anti-inflammatory properties, attenuating the release of pro-inflammatory TNF, IL-1β, IL-6 and IL-18 in LPS-stimulated human macrophage cultures, while SAM activation is known to suppress the parasympathetic nervous system, leading to reduced acetylcholine release [124,125]. (3) Activation of α-adrenoceptors in macrophages augments the immune response by increasing production of proinflammatory TNF-α [126]. Furthermore, activation of the β_2_-adrenoceptor with adrenaline increases proinflammatory IL-6 production in macrophages [127]. (4) Chronic stimulation of the β-adrenoceptor via SAM activation leads to down-regulation of the receptor leading to adaptive changes [128]. Glucocorticoids also have the capacity to modulate immune function. (5) Glucocorticoids inhibit phospholipase A_2_ which limits the conversion of cell membrane phospholipids to arachidonic acid and then into proinflammatory eicosanoids [118]. (6) Activation of the glucocorticoid receptor causes modulation of transcription of glucocorticoid response elements which have an immunosuppressive effect [115]. (7) Glucocorticoid receptor activation disrupts NFκB coactivator activity and prevents NFκB-mediated activation of inflammatory genes [120]. (8) Chronic HPA axis activation leads to glucocorticoid resistance and adaptive changes in immune function [120]. Chronic stress can also lead to adaptive immune changes through (9) ageing of the immune system [129] and (10) epigenetic modification of transcription of immune- and stress-related genes [130]. Stress also leads to neuronal activation in fear and threat appraisal centres in mouse stress models (marked by increased cFos and ∆FosB) which coincides with microglial activation. This neuronal and immune activation also recruits peripheral immune cells into the CNS, leading to exacerbation of existing neuroinflammation [50]. Created with BioRender.com.

The main physiological pathways through which stress, a potent environmental predisposing factor, modulates immune function are the sympathetic–adrenal–medullary (SAM) and the HPA axes [113]. These systems are crucial in our understanding of the impact of stress on the immunological phenotype observed in depressed patients. Exposure to stress is associated with the release of corticotropin releasing hormone from the paraventricular nucleus (PVN) of the hypothalamus which in turn activates the pituitary to release adrenocorticotophic hormone (ACTH) [114]. This in turn leads to downstream secretion of glucocorticoids from the adrenal glands. Glucocorticoids are naturally immunosuppressive and anti-inflammatory (see Figure 2) [115]. Among the anti-inflammatory genes that glucocorticoid receptors up-regulate is mitogen-activated protein kinase phosphatase-1, which inhibits proinflammatory mitogen-activated protein kinase signalling pathways [115]. Activated glucocorticoid receptors also bind co-repressor molecules and disrupt nuclear factor kappa B (NFκB) coactivator activity, reducing histone acetylation and suppressing proinflammatory gene transcription. This antagonism is also mutual, meaning that during states of chronic stress NFκB can mediate glucocorticoid resistance [116,117]. Glucocorticoids also inhibit phospholipase A_2_ and cyclo-oxygenase, which limits the conversion of arachidonic acid into proinflammatory eicosanoids [118]. Alternatively, chronic stress can lead to glucocorticoid resistance. Glucocorticoid resistance causes immune dysregulation from which an inflammatory-like phenotype may emerge. Chronic stress is associated with lower morning cortisol levels, greater afternoon cortisol levels and higher daily cortisol output [119]. Chronic stress and glucocorticoid resistance leads to a reduced cortisol response in the dexamethasone suppression test [119] (for a review of glucocorticoid resistance see Barnes & Adcock, 2009 [120]). The main mechanisms of glucocorticoid resistance include (1) agonism of the glucocorticoid receptor leading to homologous downregulation [117,121], and (2) expression of the inactive β isoform of the glucocorticoid receptor, leading to a dampened glucocorticoid response [117]. Expression of this isoform is induced by proinflammatory cytokines so it is likely that this form of glucocorticoid resistance stems from signalling within the immune system [117]. These mechanisms of glucocorticoid resistance play important roles in stress-related inflammation and are believed to mediate predisposition to depression and other stress-related disorders.

The SAM axis provides an alternative mechanism through which stress elicits effects on the immune system. During stress, sympathetic nerves secrete catecholamine neurotransmitters (noradrenaline and adrenaline) to stimulate the body’s ‘fight or flight’ response. Noradrenaline and adrenaline immunoregulate by modulating adrenergic receptors on peripheral immune cells (Figure 2) [131]. The role of β-adrenergic signalling in mediating immune responses to stress and inflammation has also been described elsewhere [126]. Wohleb et al. (2011) initially described the efficacy of the β-adrenergic receptor antagonist propranolol to reduce stress-induced anxiety-like behaviours in mice. This was accompanied by attenuation of activated microglia morphology [127]. Agonism at the β_2_-adrenoceptor can also have an anti-inflammatory effect [132]. While the HPA and SAM axes are two of the more significant physiological mechanisms involved in stress related immunomodulation, other signalling peptides are involved in regulating immune function including growth hormone, prolactin and thyroxine [133]. See Webster, Marketon & Glaser (2008) for a detailed review on stress hormones and their effects on immune cells [134].

Glucocorticoid and mineralocorticoid receptors are highly expressed in key limbic structures involved in mood and behaviour such as the hippocampus, prefrontal cortex and amygdala, rendering them highly sensitive areas to changes in HPA axis activity [135]. Glucocorticoids probably mediate stress-induced maladaptive behaviours by causing dendritic atrophy and loss of synaptic connections in key limbic structures of the brain including the prefrontal cortex and hippocampus [136]. This effect is likely to be mediated via changes in BDNF expression (discussed later). The effect of corticosteroids on plasticity in the hippocampus appears to be dose dependent and follows an inverted u-shaped relationship, with higher concentrations leading to impairment, low-to-moderate concentrations facilitating enhancement and absence again causing impairment of long-term potentiation [137]. Glucocorticoid receptor binding in the basolateral amygdala is believed to effect memory of threatening stimuli. This was demonstrated by Roozendaal & McGaugh (1997) who showed that infusion of a glucocorticoid agonist into the basolateral amygdala of rats improved their memory of an aversive foot shock [138].

Limbic structures also modulate glucocorticoid secretion from the HPA axis itself via indirect feedback mechanisms. The hippocampus and prefrontal cortex are largely inhibitory of HPA axis activation while the amygdala is believed to increase HPA axis activation [135]. While this regulatory relationship has been proposed, there is little evidence of direct innervation of the PVN of the hypothalamus by these limbic structures. Alternatively, it seems that these structures form projections to cell populations in the basal forebrain, hypothalamus and brainstem which in turn innervate the PVN of the hypothalamus [135]. An activated immune system as a result of social defeat in mice has been consistently reported. Bergamini et al. (2018) found that chronic social defeat in mice increased lymphocytes, granulocytes, inflammatory monocytes and myeloid cells in the spleen [139]. Furthermore, expression levels of TNF-α and IFN-γ were up-regulated in splenic myeloid cells [139]. Liver expression of the kynurenine pathway enzyme genes *Tdo1*, *Tdo2*, *Ido2*, *Kynu*, *3-Hao* and inducible nitric oxide synthase levels were also increased in defeated mice [139]. In the ventral tegmental area, chronic social defeat led to an increase in microglial activation and kynurenine pathway induction of defeated mice [139]. Mice subjected to defeat also exhibited less operant responding for sucrose reward, indicating an association between stress, microglial activation and reward-based behaviour [139]. In a separate study, chronic social defeat reduced motivation as measured by performance in a progressive ratio schedule motivation task and the saccharin preference test [140]. In imaging studies on mice, defeat led to increased functional connectedness in and between regions analogous to those implicated in depression, including prefrontal cortex-amygdala, ventral hippocampus-amygdala and cingulate cortex-amygdala regions [141]. The implication of these structures again suggests that the limbic system is an important pathway that links HPA axis dysfunction to neuropsychiatric disorders. It is interesting to note that imaging techniques have found the volumes of these structures to be consistently reduced in MDD, suggesting that changes in functional connactivity and neuronal atropy may be closely linked [142].

Fonken et al. (2016) demonstrated the efficacy of HMGB-1 (a molecule secreted by immune cells in response to stress) antagonism in attenuating an exaggerated immune response to infection and resulting depression-like behaviours [143]. This study treated aged rats with BoxA (a competitive antagonist of HMGB-1) 24 h prior to a peripheral *Escherichia coli* challenge [143]. This treatment attenuated immune stimulated increases in hippocampal expression of IL-1β, TNF-α and IL-6 and deficits in pre-exposure fear-conditioning (measure of hippocampal-dependent memory of an aversive stimulus) and juvenile exploration (measure of social exploration related to sickness behaviour) [143]. Central administration of disulphide HMGB-1 primes the neuroinflammatory response in rats and causes an exaggerated LPS response in terms of hippocampal mRNA levels of NF-κBIα, IL-1β, IL-6 and NLRP3 [144]. The NLRP3 inflammasome is a protein complex that functions as part of the innate immune system by binding pathogen associated- and damage associated-molecular patterns. NLRP3 facilitates the processing and release of pro-inflammatory cytokines such as IL-1β via the activation of caspase-1 [145]. HMGB-1 is thus considered a stress-induced ‘danger molecule’ that primes microglia to immune stimuli [146]. The emergence of such signalling molecules in the stress response provide novel targets for amelioration of stress-induced neuroinflammation and associated behaviours and represent a promising avenue for future studies.

### Early life adversity

Early life adversity (ELA) encompasses traumatic or stressful experiences that occur during the perinatal period right up until early adulthood. ELA can cause persistent alterations in neuroendocrine and metabolic function leading to maladaptive behaviours and increased susceptibility to poor health later in life [147]. ELA leads to chronic activation of the HPA axis, abnormal concentrations of peripheral cytokines and dysregulation of immune cell function. Baumeister et al. (2016) found that individuals exposed to childhood trauma had elevated baseline levels of C-reactive protein, IL-6 and TNF-α [148]. White blood cells cultured from adolescents living in adverse family environments also have an exaggerated IL-6 response to LPS stimulation [149]. The underlying mechanisms for this ELA immune phenotype are believed to arise from (1) abnormal glucocorticoid signalling induced by chronic activation of the HPA axis, (2) immunosenescence (ageing of the immune system) and (3) epigenetic regulation of gene transcription. Dysregulation of the HPA axis and high glucocorticoid levels as a result of early life stress has been reported in animal models of pre-natal stress [150,151]. Furthermore, children exposed to maternal stress (e.g. a mother with depression) during infancy and at 4.5 years of age have been found to have higher afternoon basal cortisol levels [152].

ELA is also associated with both immunosenescence and accelerated epigenetic ageing [129]. The most commonly used marker of immunosenescence is telomere length. There are multiple studies that report an association between ELA and shorter telomere length in immune cells [153–155] (see Price et al. (2013) for review [156]). In terms of epigenetic modification, DNA methylation is the most commonly studied mechanism that modulates gene transcription. Early life stressors such as caesarean birth are associated with accelerated epigenetic ageing [157], while cortisol output and socioeconomic background are associated with increased DNA methylation. It is interesting to note that there is extensive evidence that ELA alters epigenetic methylation at various sites within the glucocorticoid receptor gene (*NR3C1*) [130, 158–160]. The *FKBP5* gene is another stress-related gene, which appears to interact with factors such as stress and adversity. FKBP5 is a co-chaperone protein, which regulates glucocorticoid receptor activity. Mikolas et al. (2019) report that carriers of the T allele of the rs1360780 polymorphism showed a positive correlation between ELA severity and CA3 hippocampal volume [161]. For an in-depth review on epigenetic mechanisms in stress-related psychiatric disorders and immune function see Klengel & Binder (2015) [162].

## Immunogenic stimulation and monoaminergic neurotransmission

Monoaminergic and serotonergic neurotransmission have long been implicated in the pathophysiology of MDD [163–165]. The neurotransmitters implicated include noradrenaline, dopamine and serotonin. Impairment of serotonergic neurotransmission in depression contributes to a wide plethora of symptoms including poor mood, changes in appetite, changes in sleep, and sexual and cognitive dysfunction [166]. A recent meta-analysis by Moncrieff et al. (2022) indicated little conclusive support for the hypothesis that decreased serotonin activity or concentrations cause depression [167]. While this does not deny the accepted efficacy of drugs that target serotonin neurotransmission in treating depression, it raises questions regarding a role for serotonin in its aetiology. Dysregulation of the noradrenaline system in depression is believed to result in low energy, attention and concentration deficits, and reduced cognitive ability [168]. Dopamine dysfunction is believed to contribute to anhedonia, reduced psychomotor speed, impaired concentration and a lack of motivation [169,170]. Deficits in motivation and motor functioning are associated with dysfunction in corticostriatal neurocircuitry, which may be a result of alterations in mesolimbic and mesostriatal dopamine [171]. Mesolimbic reward circuitry is heavily implicated in anxiety and depression symptoms with projections from the ventral tegmental area to the nucleus accumbens and amygdala of particular relevance [172–174]. Monoaminergic systems are also susceptible to neuroendocrine and neuroimmune system response [169]. For an in-depth review of glucocorticoid and monoaminergic mechanisms in stress-related psychopathology of relevance to depression, see Tseilikman et al. (2020) [175].

### Tetrahydrobiopterin dependent transmitter biosynthesis

**Figure 3:**
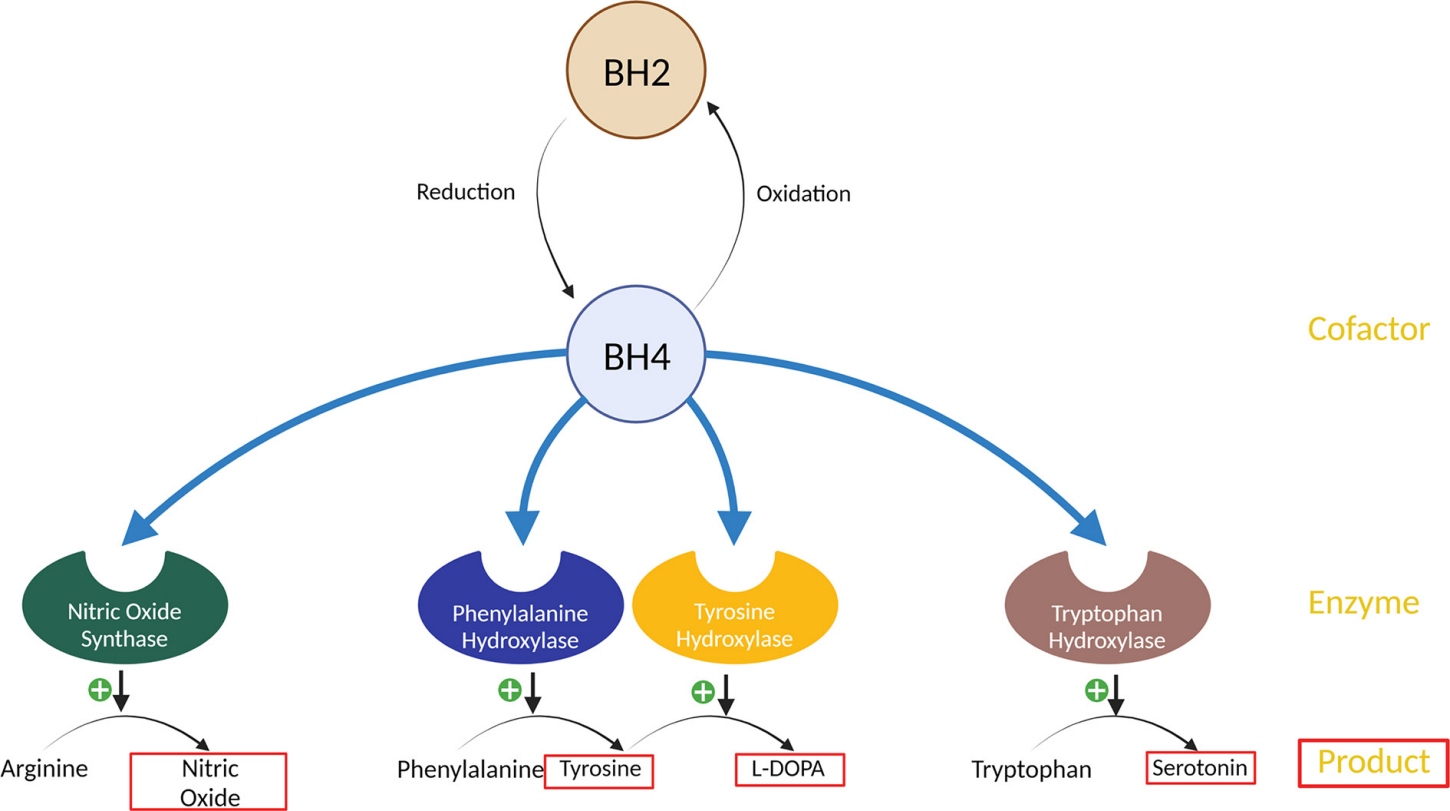
Reactions affected by tetrahydrobiopterin (BH4) availability BH4 acts as a cofactor for nitric oxide synthase, phenylalanine hydroxylase, tyrosine hydroxylase and tryptophan hydroxylase, which catalyse the synthesis of nitric oxide, tyrosine, L-DOPA and 5-hydroxytryptophan (a precursor to serotonin) respectively. Created with BioRender.com.

Inflammation impacts monoamine neurotransmission through influencing monoamine uptake, turnover and expression of neurotransmitter transporters and receptors. The relationship between monoaminergic signalling and inflammation-associated depression has been previously reviewed [176,177]. Inflammation also influences the biosynthesis of monoamines (Figure 3) [171]. Tetrahydrobiopterin (BH4) is an essential cofactor in the activity of phenylalanine hydroxylase, tryptophan hydroxylase and tyrosine hydroxylase, which synthesise tyrosine, 5-hydroxytryptophan (a precursor to serotonin) and L-DOPA (a precursor to dopamine), respectively [178]. BH4 is also a cofactor for nitric oxide synthase (NOS). Inflammation can induce NOS activity, sequestering available BH4. Nitric oxide can also contribute to oxidative stress and formation of reactive oxygen species (ROS) [179]. BH4 itself is highly redox sensitive so this redox environment contributes to the reduction of BH4 to BH2, further limiting the amount of BH4 available for the synthesis of monoamine neurotransmitters [171]. In line with this, Kitagami et al. (2003) demonstrated that systemic injection of IFN-α in rats resulted in a reduction of dopamine and BH4 in the amygdala and raphe areas. Somewhat surprisingly, levels of nitrite and nitrate (metabolites of nitric oxide, the metabolic product of NOS) were also reduced in these brain regions. It is hypothesised that an initial increase in nitric oxide by iNOS-expressing cells reduces BH4 availability as a cofactor for NOS, eventually leading to a reduction of nitric oxide levels. Administration of N^G^-monomethyl L-arginine (a nitric acid synthase inhibitor) rescued these IFN-α-induced deficits [180].

As phenylalanine is converted to tyrosine by phenylalanine hydroxylase, the phenylalanine/tyrosine ratio is an indirect marker of BH4 activity (ratio is inversely proportional to activity). This ratio is often correlated with indicators of inflammation [181,182]. IFN-α treatment in hepatitis C patients increases plasma phenylalanine/tyrosine ratios (meaning BH4 activity is reduced) when compared with patients awaiting cytokine therapy [183]. Additionally, cerebrospinal fluid BH4 levels were negatively correlated with cerebrospinal fluid IL-6 concentrations in IFN-α-treated patients [183]. A more recent study in mice demonstrated that LPS challenge reduced striatal dopamine levels and increased the phenylalanine/tyrosine ratio [184]. Altogether, this implies that inflammation diminishes BH4 activity [178]. Reduced BH4 availability limits the conversion of phenylalanine to tyrosine and of tyrosine to L-DOPA, leading to reduced levels of dopamine in the CNS likely contributing to symptoms of fatigue, anhedonia and a lack of motivation.

A single acute peripheral injection of BH4 is sufficient to increase the levels of biopterin in the brain, without impacting endogenous gene expression of proteins involved in BH4 synthesis (e.g., dihydrofolate reductase; the enzyme that catalyses the conversion of BH2 to BH4) [185]. Exogenous BH4 also increased amphetamine-induced dopamine release in the nucleus accumbens and improved performance of mice in a motivational task [185]. BH4 supply also rescues LPS-induced reductions in striatal BH4 levels, striatal dopamine concentrations and up-regulation of IL-1β and TNF-α mRNA in the striatum in mice [184]. LPS-induced deficits in locomotor response to amphetamine is also restored, suggesting that BH4 treatment modulates dopaminergic neurotransmission with potential efficacy in amotivational syndromes [184].

## Mesolimbic dopaminergic transmission

The mesolimbic dopamine reward circuit is implicated in depression. Brain imaging techniques are used to find associations between inflammatory markers and functional connectivity within the mesolimbic reward circuit. Felger et al. (2016) used resting-state functional magnetic resonance imaging in a cohort of depressed patients to relate brain connectivity to inflammation [186]. Plasma C-reactive protein concentration, a common marker for inflammation, associated with decreased connectivity between the ventral striatum and the ventromedial prefrontal cortex as well as with anhedonia [186]. Additionally, increased C-reactive protein associated with decreased connectivity between the dorsal striatum, ventromedial prefrontal cortex and presupplementary area, which correlated with decreased motor speed [186]. This study also demonstrated that connectivity between striatum and ventromedial prefrontal cortex associated with increased IL-6, IL-1β and IL-1 receptor antagonist in the plasma, providing further evidence for an association between mesolimbic network connectivity, inflammation and anhedonic behaviour [186].

Other studies have utilised animal models to elucidate the relationship between inflammation, mesolimbic reward circuitry and depressive-like behaviours. Felger et al. (2013) found that IFN-α administration to nonhuman primates decreased dopamine release in the striatum, dopamine D_2_ receptor binding and effort-based sucrose consumption [187]. Administration of the dopamine precursor, L-DOPA, reverses these reductions in striatal dopamine release, suggesting that the inflammatory stimulus reduces the availability of dopamine precursors independently of end-product synthesis, vesicular packaging and release [188]. Mice subjected to chronic social defeat have increased levels of TNF-α in the plasma and spleen accompanied by adrenal hypertrophy [189]. Gene expression in inflammation-related pathways were also altered while key genes in dopamine function including those encoding dopamine receptor 2 (*Drd2*) and dopamine and cAMP regulated phosphoprotein 32 (*Darpp-32*) were down-regulated [189], highlighting the interconnected relationship between stress, inflammation and dopaminergic signalling.

Wang et al. (2018) found that LPS induced reductions in dopamine D_3_ receptor expression in mesolimbic regions of the mouse brain, and in BDNF expression in the ventral tegmental area and medial prefrontal cortex. This was accompanied by depression-like behaviour in the forced swim and tail suspension tests [190]. Pre-treatment with pramipexole, a DR3-selective agonist, reduced LPS-induced depressive behaviours and attenuated LPS-induced increases in TNF-α, IL-1β and IL-6 in the ventral tegmental area and nucleus accumbens [190]. Pramipexole also rescued LPS-induced reductions in BDNF expression in the ventral tegmental area. In contrast, treatment with DR3-specific antagonist NGB 2904 alone increased levels of proinflammatory cytokines (TNF-α, IL-1β and IL-6) in the medial prefrontal cortex and nucleus accumbens, increased immobility in the forced swim test and reduced levels of BDNF in the medial prefrontal cortex when compared with vehicle treated control mice [190]. A study in mice subjected to LPS-induced sickness and depression-like behaviours found that leptin (an appetite suppressant) mediates an antidepressant effect by increasing BDNF levels in the hippocampus, reducing depressive-like behaviours and reversing LPS-induced alterations in IL-1β in the prefrontal cortex and striatum. Blockade of the D_1_ and D_2_/D_3_ dopamine receptors blocked these antidepressant effects, suggesting that leptin partially elicits an antidepressant effect via the dopamine receptors [191].

## A role for the kynurenine pathway

**Figure 4:**
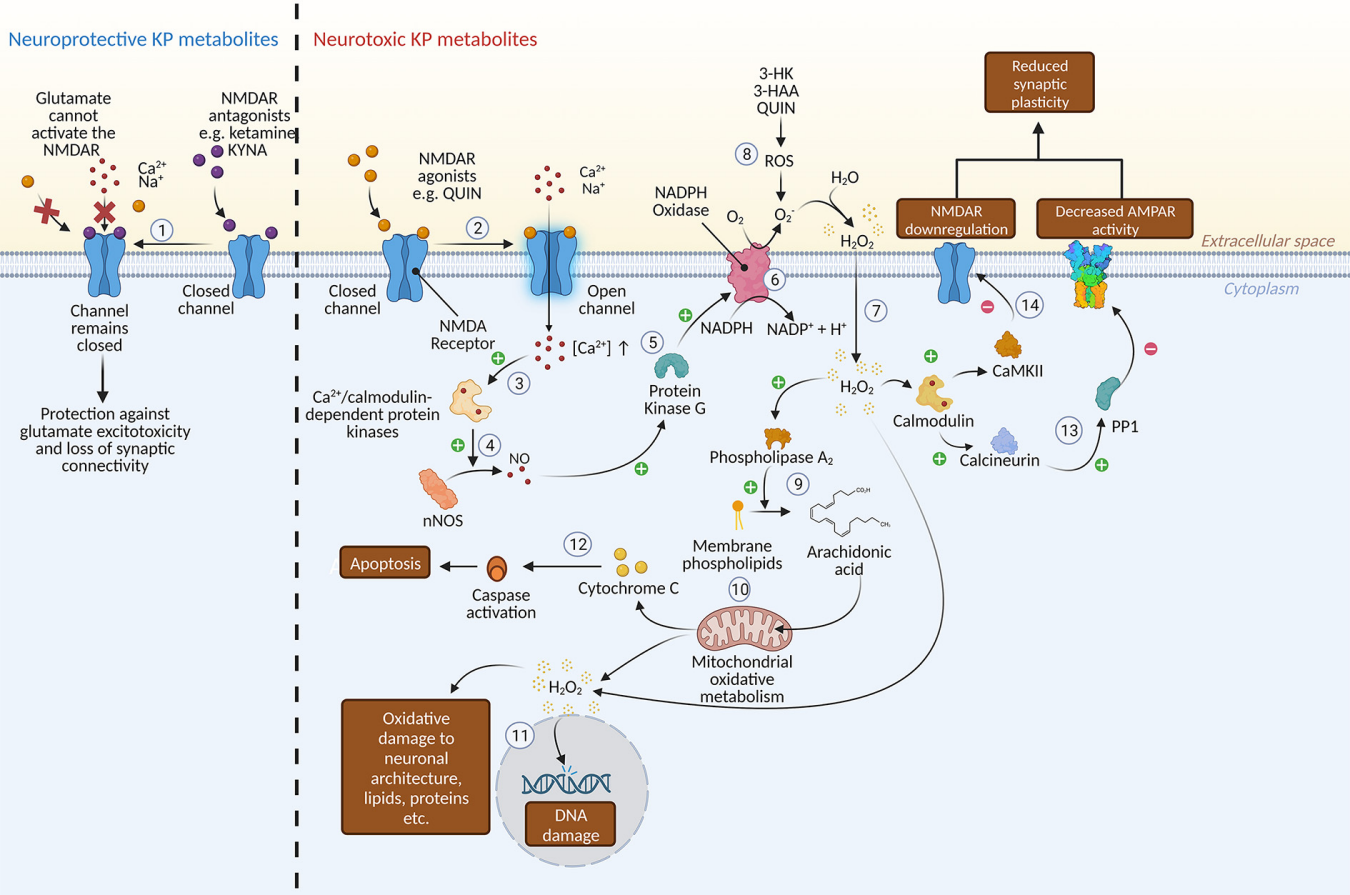
Action of KP metabolites at the NMDA receptor (1) Binding of NMDA receptor antagonist KYNA prevents activation of the NMDA receptor and subsequent calcium influx. (2) Binding of NMDA receptor agonists glutamate or QUIN activate the receptor, causing the ion channel to open (if the membrane is also depolarised) and facilitating the flow of Ca^2+^ into the cell. (3) Increase in intracellular Ca^2+^concentration activates Ca^2+^/calmodulin-dependent protein kinases (CaMK) which in turn (4) regulate the activity of neuronal NOS (nNOS) which produces NO [204]. (5) Downstream nNOS-dependent activation of protein kinase G (PKG) increases activity of NADPH oxidase in the cell membrane [205]. (6) NADPH oxidase activity in turn mediates superoxide production [205]. (7) Superoxide reacts with water to form hydrogen peroxide which passes through the membrane and accumulates intracellularly [206]. (8) Oxidative KP metabolites also generate ROS which further enhance the production of hydrogen peroxide. (9) Accumulated hydrogen peroxide in turn has the capacity to activate cytosolic phospholipase A_2_, increasing the production of arachidonic acid [207]. (10) Mitochondrial metabolism of arachidonic acid results in the formation of additional superoxide, hydrogen peroxide and cytochrome *c*. (11) Hydrogen peroxide causes oxidative damage to cellular molecules including lipids, proteins and DNA, leading to cell death. (12) Cytochrome *c* activates cellular caspases resulting in apoptosis. (13) Hydrogen peroxide facilitates the calmodulin-mediated dephosphorylation of calcineurin and protein phosphatase 1 (PP1) leading to decreased AMPA receptor activity [206]. (14) Calmodulin also dephosphorylates CaMKII, leading to downregulation of the NMDA receptor. Abbreviations: 3-HK, 3-hydroxykynurenine; 3-HAA, 3-hydroxyanthranillic acid; QUIN, quinolinic acid. Created with BioRender.com.

The kynurenine pathway (KP) is the dominant form of tryptophan metabolism in the body, with approximately 95% of tryptophan being metabolised via the KP [192]. Activation of the KP is mediated by tryptophan-metabolising enzymes indolamine 2,3 dioxygenase (IDO) and hepatic tryptophan 2,3-dioxygenase (TDO) [193]. These convert tryptophan (the serotonin precursor) to kynurenine. At this point, the KP bifurcates into one neuroprotective and one neurotoxic branch. Under the neuroprotective branch, kynurenine aminotransferase (KAT) enzymes, which are predominantly expressed in astrocytes in the CNS, convert kynurenine into kynurenic acid (KYNA). KYNA is an N-methyl-D-aspartic acid (NMDA) receptor antagonist and has well-established neuroprotective properties [193]. Alternatively, kynurenine is converted by kynurenine monooxygenase (KMO), which is mainly expressed by microglia, into 3-hydroxykynurenine (3-HK) and subsequently metabolised into 3-hydroxyanthranillic acid (3-HAA) and quinolinic acid (QUIN) [193]. 3-HK and 3-HAA elicit a neurotoxic effect through the generation of free radicals while QUIN is both oxidative and an NMDA receptor agonist, capable of causing excitotoxicity (see Figure 4) [193]. The blood–brain barrier is another key player in KP compartmentalisation. KYNA cannot cross the BBB, so increasing the conversion of kynurenine to KYNA in the periphery restricts the central supply of kynurenine, limiting production of neurotoxic metabolites in the brain [193]. Alterations in KP metabolism has been implicated in a range of CNS disorders including depression [193], bipolar disorder [194–196], schizophrenia [197], neurodegenerative disease [197] and sleep disturbances [198]. KP disturbances have also been shown to associate with changes in hippocampal subfield and striatal volumes in depressed patient cohorts [199,200]. The role of altered KP metabolism in depression has been reviewed previously [1,193,197,201–203]. While KP metabolism favouring neurotoxicity is often implied in depression, the evidence in support is inconclusive [193].

### Induction of the kynurenine pathway

Inflammation and stress are the predominant mechanisms by which the KP is induced. Immune stimuli such as the viral mimetic polyinosinic:polycytidylic acid (polyIC) increase the levels of central and peripheral proinflammatory cytokines and induce expression of IDO in the rat brain [208]. In their review, Salazar et al. (2012) proposed that induction of IDO and resulting KP activation by LPS stimulation induces a depressive phenotype [209]. This phenotype is attenuated by the IDO inhibitor 1-methyltryptophan [209] or genetic deletion of the *ido* gene [210]. Glucocorticoids also induce hepatic TDO leading to KP activation in the periphery [211]. TDO increases conversion of tryptophan into kynurenine which can in turn cross the blood–brain barrier and be converted into neurotoxic KP metabolites depending on the pathway induction/activation state within the CNS [212]. Accordingly, it has been proposed that KP activation initiated by stress-induced stimulation of hepatic TDO also contributes to a depressive phenotype [213]. Similarly to the immune stimulated model of depression, stress-induced alterations in anxiety- and depression-like behaviours are attenuated by TDO inhibition with allopurinol [213] and deletion of the TDO gene [214].

Systemic LPS administration leads to a deficit in recognition memory in mice accompanied by an increase in brain and plasma kynurenine (biomarker of KP activation); however, these LPS-induced deficits in recognition memory are not present in *ido*^−/−^ and *kmo*^−/−^ mice [215]. Memory deficits seen here may be related to cognitive dysfunction seen in inflammation-induced depression. Similar investigations were undertaken in *kmo*^−/−^ and *haao*^−/−^ mice [216]. 3-Hydroxyanthranilic acid dioxygenase (HAAO) is an enzyme downstream of KMO in kynurenine metabolism that generates neurotoxic QUIN. LPS challenge results in depression-like behaviours in the tail suspension, sucrose preference, open field and Y-maze tests in mice [216]. In this particular study, both *kmo*^−/−^ and *haao*^−/−^ mice were protected from LPS-induced deficits in the tail suspension and Y-maze tests [216]. Furthermore, administration of 3-HK (the initial product of KMO metabolism of kynurenine) induced depressive behaviours in *kmo*^−/−^ mice in a dose-dependent fashion [216], supporting the idea that ‘neurotoxic’ KP metabolism is a key mediator of these depressive behaviours.

Recent studies have attempted to characterise KP metabolism in a brain region-specific manner. For example, Parrott et al. (2016) identified the dorsal hippocampus as a brain region in mice that is particularly vulnerable to LPS-induced activation of the KP [216]. This brain region had higher concentrations of 3-HK, 3-HAA and xanthurenic acid (a 3-HK metabolite) while KYNA concentrations were unaffected [216]. The other brain regions investigated (ventral hippocampus, central amygdala and nucleus accumbens) did not show upregulation to the same extent, suggesting that the dorsal hippocampus is a region of particular vulnerability in terms of ‘neurotoxic’ KP metabolism [216]. This is a particularly interesting finding as it suggests that KP induction may directly contribute to reduced hippocampal neurogenesis, which is commonly implicated in depression.

The relationship between KP metabolism and selected cell signalling pathways has been further studied. Cathomas et al. (2015) investigated a role for CD40 signalling in KP activation and sickness behaviour syndrome [217]. CD40 is a transmembrane protein required for the activation of antigen-presenting cells. CD40 agonist antibody administered to mice led to decreased saccharin drinking, decreased fear conditioning and KP activation in the brain and periphery [217]. These effects were dependent on TNF-α signalling as co-administration of TNF-α blocker etanercept with CD40 agonist antibody prevented the onset of depressive-like behaviours and restored levels of KP metabolites to normal [217]. Co-administration of an undisclosed novel IDO1 inhibitor decreased production of KP metabolites kynurenine, QUIN, KYNA and 3-HK but did not reverse sickness behaviours, suggesting that KP induction occurs as a result of CD40-TNF activation but pathway metabolites are not necessary for the CD40-TNF-α-induced initiation of sickness and depression-like behaviours [217].

Multiple KP metabolites have affinity for the NMDA receptor, providing a possible mechanism whereby KP activation induced by inflammation and/or stress regulates synaptic plasticity [211]. Within the CNS, QUIN is a potent NMDA receptor agonist, meaning it can have effects similar to that of glutamate. QUIN-mediated excitotoxicity can ensue as a result of NMDA receptor overactivation (see Figure 4). Treatment of rat primary striatal neurons with QUIN causes hyperphosphorylation of neurofilaments [218] and reduces the number and outgrowth of neurites *in vitro* [218,219]. Injection of QUIN into the medial prefrontal cortex of mice induced an initial increase in hippocampal long-term potentiation followed by a gradual impairment over the subsequent 14 days [220]. QUIN initiates changes in the neuronal cytoskeleton leading to synaptic and neuronal cell loss. These alterations coincided with behavioural and cognitive deficits in a reversal variant of the Morris water maze task [220].

KYNA functions as an antagonist at the glycine site of the NMDA receptor [221]. Additionally, KYNA inhibits glutamate release in the CNS, since oral administration of BFF816 (a KATII inhibitor which limits KYNA synthesis) attenuates inhibition of glutamate release in the prefrontal cortex [222]. Similarly, KATII knockout mice have increased extracellular glutamate, associated with an increase in the amplitude of long-term potentiation assessed in hippocampal slices *in vitro* [223]. As an NMDA receptor antagonist, KYNA is believed to directly counteract the effects of QUIN and protects against excitotoxicity. The KYNA/QUIN ratio at glutamate receptors is believed to be a factor in regulating excitatory postsynaptic potentials and further changes in activity mediated changes to neuronal complexity. Verstraelen et al. (2014) utilised *in vitro* calcium imaging of primary mouse neurons to demonstrate this. This study showed that KYNA reduced the frequency of synchronous bursts of action potentials which could be normalised by subsequent QUIN addition. Furthermore, QUIN was shown to have an excitatory effect on synchronous burst activity which could be reversed by subsequent KYNA addition [224]. KYNA can also rescue QUIN-induced cytoskeletal changes in mixed neuronal/glial cultures [219]. L-kynurenine sulfate, a KYNA precursor, has been shown to rescue the level and duration of long-term potentiation in a rat model of ischaemia [225]. Lower doses of KYNA (0.25 µg/µl) in mice has protective effects on memory, whereas higher concentrations (10*–*20 µg/µl) has detrimental effects on memory [226]. This is likely because at higher concentrations, KYNA begins to effect other receptors, KYNA also modulates activity of the α7 nicotinic acetylcholine receptor [227], the aryl hydrocarbon, the GPR35 and the AMPA/kainate receptors [228]. While NMDA receptor activation induces long term potentiation, it is AMPA receptor-mediated neurotransmission itself that strengthens synaptic connections. Low concentrations of KYNA facilitates AMPA receptor responses by mediating allosteric modulation of the AMPA receptor. This facilitates membrane depolarisation and increased NMDA receptor activation. At higher concentrations of KYNA, competitive glutamate receptor antagonism prevails [229].

Under physiological conditions, ROS are essential signalling molecules necessary for synaptic plasticity and cognitive function [230]. However, at pathological concentrations ROS overwhelm endogenous antioxidant mechanisms. Oxidative stress ensues, causing damage to membrane lipids, DNA molecules and proteins, leading to impaired synaptic integrity and plasticity (see Figure 4) [206]. 3-HK, 3-HAA and QUIN are three oxidative KP metabolites capable of directly producing toxic ROS [193]. As a result, it is likely that induction of the KP produces toxic concentrations of oxidative metabolites with the potential to result in ROS-induced neuronal damage.

An upregulation of KATI and KATII (which convert kynurenine to KYNA) expression is evident in anterior cingulate cortex post-mortem tissue of depressed patients [231]. Expression of astrocytic excitatory amino acid transporter 2 (EAAT2; a major glutamate transporter, responsible for 90% of total glutamate uptake [232]) is also reported to be upregulated in this brain region in depressed patients [231]. These findings suggest that depression may relate to an alteration of glutamatergic neurotransmission in this brain region since EAAT2 facilitates astrocytic glutamate reuptake, while KAT enzymes produce KYNA, an NMDA receptor antagonist. This study also implicates astrocyte pathology in depression as EAAT2, KATI and KATII are astrocyte-specific proteins [231].

Peripheral KYNA/QUIN ratios were correlated with amygdalar and hippocampal volumes, suggesting that neurotoxic KP metabolism contributes to regional atrophy, particularly in these brain regions [233]. Meier et al. (2016) used MRI in a cohort of depressed patients and healthy controls to demonstrate a negative correlation between rostral anterior cingular cortical thickness and serum levels of C-reactive protein, which was partially mediated by the KYNA/3-HK ratio [234]. These findings support a working hypothesis that KP induction, possibly as a consequence of underlying inflammation or dysregulated immune function may trigger cortical atrophy which in turn contributes to the manifestation of depression-related symptoms. Overall, the KP offers a critical link between immune activation, inflammation and the disruption to neuronal processes such as glutamatergic neurotransmission, astrocytic function, neurogenesis and grey matter volumes, all of which are commonly implicated in depression pathogenesis.

## Inflammation response and neurotrophic signalling

β_2_-Adrenoceptor activation leads to downstream activation of adenylate cyclase, production of cyclic adenosine monophosphate (cAMP), activation of protein kinase A, and phosphorylation of various transcription factors [235] including cAMP response element binding protein (CREB). CREB regulates gene transcription by binding to the promotor regions of target genes. With regard to immune function, CREB can have an anti-inflammatory effect, e.g. by inducing IL-10 and stimulating generation of Treg cells [236]. Alternatively, CREB promotes activation and proliferation of T and B cells and differentially regulates Th1, Th2 and Th17 cells [236]. CREB partially regulates the BDNF promotor [237] and the expression of BDNF, a key molecular player in neuroplastic changes involved in learning and memory [238]. Peripheral BDNF levels are consistently lower in depressed patients compared with healthy controls [239,240]. A reduction in BDNF is commonly implicated in regional atrophy [241] where depression has been associated with reduced hippocampal volume, impaired hippocampal neurogenesis, and hypoactivity of the dorsolateral prefrontal cortex [242]. The ability of corticosteroids to suppress the expression of neurotrophic and growth-associated factors in the brain has been well-established [243,244]. This is seen where exposure to stress drives a rise in circulating corticosterone leading to supressed BDNF expression in the rat hippocampus [244]. Animal models suggest that this reduction in BDNF drives maladaptive changes in neuroplasticity and neuronal atrophy. It is likely that these neuroplastic changes are linked to the reduction of brain volume in key limbic structures seen in depression (for review see Price & Duman (2020) [245]). Lakshminarasimhan & Chattarji (2012) showed that chronic immobilisation stress leads to increased levels of circulating corticosterone and reduced BDNF expression in the CA3 region of the rat hippocampus. Interestingly, this study also demonstrated chronic immobilisation stress led to an increase in BDNF expression in the basolateral amygdala [246]. This supports earlier work by this group which found that this stress caused dendritic atrophy and debranching in the CA3 pyramidal neurons and enhanced dendritic arborisation in the pyramidal and stellate neurons of the basolateral amygdala [247]. Furthermore, Makhathini et al. (2017) showed stress-induced suppression of hippocampal BDNF associated with behavioural deficits in the novel object recognition and open field tests as well as reduced methylation of the hippocampal genome [248]. Importantly, Naert et al. (2007) demonstrated that steroids such as pregnenolone and dehydroepiandrosterone modulate HPA axis activity and BDNF expression in male rats in a region-specific manner, again highlighting how neuroendocrine alterations may contribute to depression pathophysiology [249]. These findings support a working hypothesis that dysfunction of the HPA axis contributes to regional changes in neurotrophic factor expression which in turn causes changes in structural plasticity and atrophy of key limbic structures contributing to the manifestation of maladaptive behaviours.

Direct immune stimulation of rats with LPS reduces levels of BDNF protein in the hippocampus, frontal cortex, parietal cortex, temporal cortex and occipital cortex [250] and reduces BDNF mRNA in the hippocampus [251]. Rats challenged with polyIC exhibit anhedonic behaviour, and show reduced expression of BDNF and its receptor TrkB in the hippocampus and frontal cortex. This is accompanied by an increase in IL-1β, IL-6, TNF-α and CD11b (a leukocyte-specific receptor and marker for monocyte/macrophages/microglia, granulocytes, and natural killer cells) expression in these same brain regions [208]. Furthermore prior exposure to stress can exacerbate subsequent responses to immune stimulus in animal models. For example, prior exposure to tail shock stress has been shown to augment plasma levels of IL-6 in response to intraperitoneal LPS challenge in rats [252]. Subsequent studies by this group have replicated this finding and have also shown that inescapable tail shock augments hippocampal mRNA expression of IL-6, IL-1β, TNF-α and NFκBIα in response to the same stimulus [253,254]. Given the aforementioned effects on BDNF expression, the inflammatory response is a potential mechanism underlying alterations in key limbic structures seen in depression.

BDNF secretion may also be a means of providing inhibitory feedback to the immune system [255]. IL-1β, IL-6 and TNF-α are up-regulated centrally in LPS-stimulated mice that are genetically deficient in BDNF (BDNF^+/−^) when compared with wild-type control (BDNF^+/+^) [256]. This was accompanied by an increase in LPS-induced anhedonia behaviour, increased expression of IDO and increased concentrations of kynurenine, 3-HK, xanthurenic acid and QUIN in whole brain tissue [256]. It is also possible that BDNF deficiency caused by HPA axis activation increases vulnerability to anhedonia-like symptoms of depression. Dugan et al. (2015) subjected wild-type and BDNF^+/−^ mice to unpredictable chronic mild stress and measured the expression of various cytokines and KP enzymes in the brain. Stress did not induce the expression of proinflammatory cytokines in these mice [257]. However, BDNF^+/−^ mice exhibited lower mRNA expression of anti-inflammatory IL-10 in response to stress [257]. Stress also induced activation of the neuroprotective arm of the KP in wild-type mice but not in BDNF-deficient mice subjected to the same protocol (characterised by up-regulation of IDO1 and TDO, down-regulation of KMO and increased levels of neuroprotective KYNA) [257]. These findings suggests that BDNF augments the anti-inflammatory response and protective KP metabolism.

## Inflammation and depression symptoms

A growing number of studies in depression research aim to link individual symptom clusters to inflammatory biomarkers. Various depression subtypes encompass a myriad of symptom clusters with varying polarity. For example, a core symptom of melancholic depression is insomnia or poor-quality sleep while a central feature of atypical depression is hypersomnia. It is plausible that these differences in symptom presentation may be caused by immunological differences and that various cytokines contribute more heavily to particular symptoms. Anisman et al. (1999) describe differences in inflammatory and stress-related markers between patients with depression and dysthymia with typical and atypical features [258]. Their study found that plasma concentration of cortisol and adrenocorticotrophic hormone were increased in patients with atypical depression but not in those with dysthymia (typical or atypical) or in depression with a typical presentation when compared to a healthy control cohort. They also found that lymphocytes isolated from dysthymic patients (typical and atypical) secreted more IL-1β in response to mitogen stimulation and that while all subgroups secrete less IL-2 when compared with healthy control, this reduction is less profound in the group of patients with a typical symptom profile [258]. It is possible that this variability in cytokine response also drives differences in disease presentation. Yoon et al. (2012) also described alterations in cytokine response in whole blood isolated from patients with melancholic and atypical depression. Similar to the aforementioned study, this group found that stimulation of whole blood with phytohemagglutinin and lipopolysaccharide led to an increased secretion of IL-2 in patients with atypical depression when compared with those with melancholic depression [259]. This study also observed a reduced secretion of IL-4 in the atypical depression group in comparison the melancholic depression. IL-4 is regarded as an anti-somnogenic cytokine and excessive sleep is a core symptom of atypical depression [260]. Furthermore, a meta-analysis by Milaneschi et al. (2020) found that strong associations between inflammatory markers and depression only emerged when comparing the control group with depressed patients with an atypical symptom profile [261]. Despite females appearing more susceptible to the depressogenic effects of inflammation than men [262], studies investigating the sex-specific effect of certain inflammatory cytokines and individual symptom clusters is scarce and offers a promising avenue to improve existing knowledge. In order to achieve this, future preclinical and clinical research should include investigations of sex differences and emphasis should be placed in particular on the use of both males and females in preclinical animal research.

## Future directions and concluding remarks

An understanding of neuroimmune origins of depression and other stress related psychiatric disorders is growing although underlying mechanisms linking immune, endocrine and neuronal systems to behavioural and psychological symptoms are not fully elucidated. Genetically modified animals continue to enable a study of immune-related genes in pathophysiological and behavioural traits associated with depression [263–265]. There is a growing appreciation that depression is a sexually dimorphic disorder [266,267] extending to neuroimmune features [268,269]. Despite this, females are often under-represented in preclinical studies [270], a limitation which must be addressed in ongoing and future research. Insights from neuroimmune interactions have led to some novel approaches to developing new therapeutic interventions. There is a growing interest in drugs targeting cytokines and their targets as putative antidepressants [271,272]. Ketamine, a recently approved rapid acting antidepressant [273], modulates the adaptive immune response to stimulation [274–276] prompting some speculation that immunomodulation could in part account for its antidepressant effect. Inhibiting KP targets IDO [277,278], TDO [213,279] and KMO [280] have produced antidepressant effects in animal studies, favouring a KP targeted approach, meriting further research. There is a need for fluid and neuroimaging biomarkers as aids to clinical practice. For a comprehensive meta-analysis of the most prominent peripheral blood biomarkers in depression, see Carvalho et al. (2020) [281]. Among those positively associated with depression are C-reactive protein, IL-6 and TNF-α while markers negatively associated with depression include KYNA, KYNA/3HK ratio, KYNA/QUIN ratio and BDNF [281]. Inclusion of immune-related markers in biomarker panels will likely help to stratify patients into more homogenous subgroups [282]. This may in turn aid with the design of clinical trials [283], to help predict which patients are likely to respond to treatment [284,285], taking a more personalised approach to treatment [286].

## Data Availability

Not applicable

## Abbreviations

3-HAA: 3-hydroxyanthranillic acid
3-HK: 3-hydroxykynurenine
BDNF: brain-derived neurotrophic factor
CAM: cell adhesion molecule
cAMP: cyclic adenosine monophosphate
CREB: cAMP response element binding protein
NOS: nitric oxide synthase
QUIN: quinolinic acid
